# Commercial Vinegar Tablets Do Not Display the Same Physiological Benefits for Managing Postprandial Glucose Concentrations as Liquid Vinegar

**DOI:** 10.1155/2020/9098739

**Published:** 2020-12-16

**Authors:** Natasha K. Feise, Carol S. Johnston

**Affiliations:** College of Health Solutions, Arizona State University, 550 N. 3 Street, Phoenix, AZ 85004, USA

## Abstract

**Objective:**

Research evidence suggests that vinegar may effectively reduce postprandial glucose in both healthy adults and those with type 2 diabetes. There is heightened consumer interest in commercially available vinegar tablets; however, it is not known whether these products lower postprandial glycemia to the same extent as liquid vinegar. This crossover trial examined the impact of liquid vinegar versus commercial vinegar tablet ingestion at the start of a meal on the 60-minute glucose excursion postmeal in healthy adults.

**Methods:**

Twelve young men and women (22.6 ± 0.6 y; 21.2 ± 1.2 kg/m^2^) completed this 4-arm Latin square crossover trial. Testing was separated by one week and consisted of a test meal (64 g carbohydrate) consumed immediately following one of the four oral treatments: CON, 60 g water (control treatment); VIN, 25 g liquid vinegar (5% acidity; 1.25 g acetic acid) diluted with 35 g water; PILL, four vinegar tablets (1.50 g acetic acid) swallowed whole with 60 g water; and PILL-c, four crushed vinegar tablets dissolved in 60 g water. Capillary blood glucose was tested in the fasted state and at 30 and 60 minutes postmeal.

**Results:**

The 60-minute glucose excursion varied significantly by treatment (iAUC: 4.9 ± 0.6, 3.4 ± 0.4, 4.9 ± 0.6, and 4.1 ± 0.5 mmol˖h/l for CON, VIN, PILL, and PILL-c, respectively; *F* (3, 33) = 3.037, *p* = 0.043; repeated measures ANOVA). Post hoc analysis revealed a 31% reduction in the glucose postmeal excursion for VIN in comparison to CON and PILL (*p* = 0.040 and *p* = 0.049, respectively).

**Conclusions:**

These data suggest that commercial vinegar tablets taken whole at mealtime are not as effective as liquid vinegar for reducing the postmeal glucose excursion in young, healthy adults.

## 1. Introduction

Worldwide, diabetes represents the fastest growing chronic disease condition affecting over 460 million adults, including 20% of adults over 65 years of age [1]. Type 1 diabetes, an autoimmune condition that destroys the beta cells of the pancreas leaving patients unable to produce insulin, typically manifests in the first 20 years of life but accounts for less than 10% of diabetes cases. The majority of cases are type 2 diabetes (T2D), characterized by an insulin resistance linked to both genetic and environmental factors. Hence, elevated blood glucose, both fasting and postprandial, is the classic diagnostic criteria for diabetes, and when left unchecked, elevated blood glucose concentrations lead to many of the complications linked to the diabetic condition including heart disease, stroke, kidney damage, and nerve damage [1, 2]. Although effective oral hypoglycemic agents are available to treat T2D [2], patients are eager for simple, safe, and affordable strategies to help manage their condition in concert with their medications. Vinegar has received much attention in recent years as an adjunct therapy for T2D, and empirical evidence is mounting demonstrating its efficacy.

Vinegar ingestion at mealtime alters postprandial glucose excursions, and recent reviews and meta-analyses suggest that vinegar may be a useful adjunct therapy for managing postprandial glycemia [3–5]. The latter meta-analysis pooled data from 11 trials and demonstrated a significant mean reduction in postprandial glycemia for the vinegar treatment compared to the control treatment (SMD = −0.60, 95% CI −1.08 to −0.11, *p* = 0.01). Although the mechanisms of action are uncertain, the defining ingredient of vinegars, acetic acid, when consumed at mealtime, appears to interact with disaccharidases in the small intestine to slow glucose entry into blood, to decrease the rate of gastric emptying, and/or to hasten blood glucose uptake into skeletal muscles [3, 6–8].

Daily vinegar ingestion (∼2 tablespoons daily for up to 12 weeks) has also been demonstrated to reduce fasting plasma glucose and insulin concentrations, as well as hemoglobin A1c values, in individuals with T2D and those at heightened risk for T2D. Gheflati et al. recently reported a significant reduction in both fasting blood glucose (−7%) and insulin (−42%) concentrations in patients with T2D after eight weeks of daily vinegar ingestion; in comparison, the control patient group displayed a 12% increase in fasting glucose concentrations during the trial [9]. In healthy individuals with a heightened risk for T2D, daily vinegar ingestion for 12 weeks significantly reduced fasting blood glucose concentrations up to 13%, changes that achieved statistical significance from the comparison control groups [10, 11]. In a 12-week randomized controlled trial in individuals with T2D, daily vinegar ingestion significantly reduced hemoglobin A1c in comparison to the control group (−0.16 units versus +0.06 units, respectively, *p* = 0.003) [12]. Although vinegar ingestion promotes modest but physiologically relevant benefits for both patient and at-risk populations, liquid vinegar consumption is not well tolerated and can invoke nausea [13, 14].

Consumer trends indicate a rise in commercial vinegar tablet usage, and it is possible that consumers consider vinegar tablets an alternate for liquid vinegar for managing blood glucose. Given the low acetic acid content of most vinegar tablets, the tablet form of vinegar does not possess the same glucose lowering effect as the liquid form and has proven to be a suitable control tablet in randomized controlled trials studying the effects of liquid vinegar ingestion [10, 11]. However, a formal trial to compare the glucose lowering properties of commercial vinegar tablets with that of liquid vinegar has not been conducted. We hypothesize that liquid vinegar consumed at mealtime (1.25 g acetic acid), in comparison to vinegar tablet ingestion (1.50 g acetic acid), will be more effective for reducing the postmeal excursion in blood glucose in healthy adults.

## 2. Methods

Healthy, nonsmoking men and nonpregnant women (18–35 y) were recruited from a campus population using electronic advertisements and flyers. Participants were free from unresolved health conditions, chronic disease including diabetes, and/or gastroesophageal reflux disease or other oral pathologies. All participants provided written consent, and the study was approved by the Institutional Review Board at Arizona State University (STUDY00006781).

The study followed a 4-arm Latin square crossover design with each participant serving as their own control and one week separating treatment days. On the day prior to testing, participants restricted moderate-to-vigorous activities and avoided caffeine and alcohol. Also, to minimize the known influence of recent macronutrient intake on postprandial glycemia testing [15, 16], participants ingested one bagel at the lunch meal and a second bagel at the dinner meal on the day prior to testing. Plain bagels (54 g carbohydrate and 300 kcal; Chompie's Baking Co., Phoenix, AZ) were provided to participants. Participants fasted overnight (10 h) and presented at the test facility in a rested state. Following fasting blood glucose measurement, participants ingested the liquid or pill treatment immediately prior to consuming the test meal. The test meal was composed of juice (6.75 oz.; 14 g carbohydrate and 60 kcal; Harvest Hill Beverage Company, Stamford, CT) and a toasted plain bagel (Chompie's Baking Co., AZ) sliced in half and topped with 30 g strawberry jam (9.5 g carbohydrate and 75 kcal; Smucker's, Orrville, OH). Participants finished the test meal within 10 minutes under observation. At the first bite of the meal, a timer was set and blood glucose was measured at the 30- and 60-minute mark. Participants remained seated and were allowed to drink only water during the postprandial testing period.

The treatments were composed of liquid vinegar (apple cider vinegar, Bragg, Santa Barbara, CA) or commercially available vinegar tablets (Nature's Life, Park City, Utah). There were four treatments: CON, 60 g water (control treatment); VIN, 25 g liquid vinegar (5% acidity; 1.25 g acetic acid) diluted with 35 g water; PILL, four vinegar tablets (1.50 g acetic acid based on label information) swallowed whole with 60 g water; and PILL-c, four crushed vinegar tablets dissolved in 60 g water. The amount of liquid vinegar that has been reported to have significant antiglycemic effects ranges from 10 to 28 g (2 teaspoons to 2 tablespoons) equating to 0.5–1.7 g acetic acid [17–19].

Capillary blood glucose from a finger prick was measured using a portable glucose check monitor (Accu-Chek Advantage Blood Glucose Monitoring System, Roche Diagnostics, Indianapolis, IN). Participants were assigned a specific monitor during the study, and all monitors were calibrated in the morning before testing according to manufacturer instructions.

Data (mean ± SE) were analyzed using the Statistical Package for Social Sciences (SPSS) 23.0 (SPSS, Inc., Chicago, IL, USA) with significance set at *p* < 0.05. Power calculations based on previous studies indicated that 10 participants would provide over 90% power to detect a 20% change in postprandial glycemia [17, 18]. The incremental area-under-the-curve (iAUC) was calculated using the trapezoidal rule. iAUC data were log transformed to normalize based on the Shapiro–Wilk test for normality, and pairwise treatment effects were examined using repeated measures ANOVA following a significant F-test.

## 3. Results

Fifteen adults enrolled in the study, and 12 participants (5 men; 7 women) completed all four treatments. The participants that withdrew cited time conflicts (*n* = 2) and dislike of vinegar (*n* = 1) for discontinuing the study. On average, participants were young and normal weight (22.6 ± 0.6 y; 21.2 ± 1.2 kg/m^2^). Demographic variables did not correlate to fasting glucose or to the 60-minute glucose excursions during the trial.

**Figure 1:**
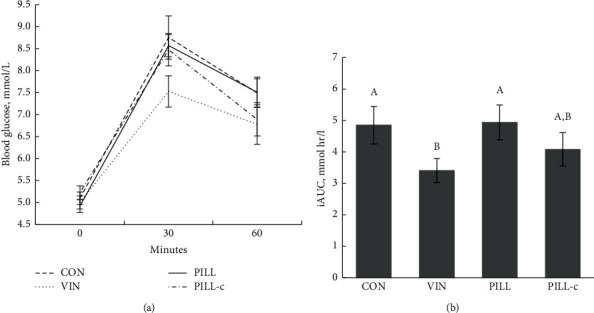
(a) Line graph representing the 60-minute postprandial flux in blood glucose under four test conditions: CON, 60 g water (control treatment); VIN, 25 g liquid vinegar (1.25 g acetic acid) diluted with 35 g water; PILL, four vinegar tablets (1.50 g acetic acid) swallowed whole with 60 g water; and PILL-c, four crushed vinegar tablets dissolved in 60 g water (*p* = 0.089; repeated measures ANOVA). (b) The blood glucose incremental area-under-the-curve (iAUC) for the four test conditions (*p* = 0.043; repeated measures ANOVA). Columns that do not share a common superscript differ significantly (*p* < 0.05).

For the four trials, fasting glucose averaged 5.06 ± 0.11 mmol/L (mean range: 4.4–5.6 mmol/L), and there were no differences in the weekly mean fasting glucose concentrations during the 4-week trial (*F* (3, 33) = 1.288, *p* = 0.295; repeated measures ANOVA). Furthermore, the order of treatment did not significantly impact fasting glucose concentrations. The glucose responses at 0, 30, and 60 minutes postmeal are shown in Figure 1(a), and the time*∗*treatment interaction did not attain significance, *F* (6, 66) = 1.926, *p* = 0.089. The 60-minute glucose excursion varied significantly by treatment (iAUC: 4.9 ± 0.6, 3.4 ± 0.4, 4.9 ± 0.6, and 4.1 ± 0.5 mmol + h/L for CON, VIN, PILL, and PILL-c, respectively; *F* (3, 33) = 3.037, *p* = 0.043; repeated measures ANOVA). Post hoc analysis revealed that 31% reduction in the glucose postmeal excursion for VIN in comparison to CON and PILL was significant (*p* = 0.040 and *p* = 0.049, respectively); however, the glucose postmeal excursions for VIN and PILL-c excursions did not differ significantly (*p* = 0.278; Figure 1(b)).

## 4. Discussion

Although the antiglycemic effect of liquid vinegar is well documented in the postprandial period in both healthy adults and those with T2D [7, 8, 17–19], the data herein suggest that commercial vinegar tablets when ingested whole do not possess this antiglycemic effect. The acetic acid content of the two vinegar preparations was comparable and within the reported range of effectiveness, 0.5–1.7 g. Reasons for these discrepant results are not clear. The dissolution rate of the vinegar tablets is not provided by the manufacturer and may factor in these results. The postmeal glucose excursion noted for the PILL-c trial arm (when the pills were pulverized and dissolved in water prior to ingestion) fell midway between that recorded for the CON and VIN trial arms (ns). This is suggestive of a slow dissolution rate for the tablet. Hence, vinegar tablets may possess antiglycemic effects if dissolved in water prior to ingestion.

The stability of acetic acid in the tablet matrix may also factor into these results and help to explain the lack of an antiglycemic effect for vinegar tablets. It is important to note that the antiglycemic effect of acetic acid is not apparent for its acetate ion. Brighenti et al. demonstrated that although liquid vinegar providing 1 g acetic acid reduced postprandial glycemia by 30% in healthy adults, postprandial glycemia was not impacted by ingestion of the same amount of neutralized vinegar (e.g., a stoichiometric equivalent amount of sodium bicarbonate was added to the native vinegar to convert the acetic acid to sodium acetate) [20]. However, both acetic acid and acetate salts effectively raise blood acetate levels. Sugiyama et al. [21] demonstrated that blood acetate concentrations were similar following ingestion of liquid vinegar or vinegar tablets (each preparation contained 750 mg acetate) based on the 180-minute area-under-the-curve indexes; however, the peak acetate concentration was 62% higher for the liquid vinegar (*p* < 0.001). Although blood acetate concentrations are comparable between the liquid and tablet vinegars, the stated conclusion of Sugiyama et al. that ingesting vinegar tablets is “as useful as the vinegar drink for achieving the health benefits of vinegar,” needs further validation as indicated by the data reported herein.

Study limitations include the small sample size, but the crossover design reduced between-subject variability and represented a study strength. As this study focused on the glucose excursion in healthy participants, data were collected only for the first hour postmeal; hence, data interpretation is limited to immediate postmeal glycemia in healthy young adults. Furthermore, capillary blood acquired via fingerstick was utilized to determine the glycemic responses as recommended by Wolever et al. since glucose concentrations in venous blood are generally lower and more variable [22]. However, this strategy limited further analyses to gain greater insights into mechanisms such as insulin analyses. Finally, we relied on the product labels to provide the acetic acid content of the vinegar products and did not determine the acetic acid content in a laboratory.

Postprandial glycemia is directly related not only to hemoglobin A1c concentrations and to diabetes risk but also to endothelial damage and cardiovascular disease risk [23, 24]; thus, healthy individuals also benefit from reductions in postprandial glycemia. Levitan et al. [25] conducted a meta-analysis of 29 studies (encompassing 194,658 adults without known diabetes followed for 12 years) and demonstrated a 36% increased risk of cardiovascular disease in adults placed in the highest versus lowest group for postprandial glycemia. In a second meta-analysis incorporating 14 studies averaging a 10-year follow-up, Kodama et al. [26] noted a dose-response relationship between postprandial glycemia and cardiovascular disease risk in the sample (*n* = 70,889), representing both healthy adults and adults with T2D. In this report, the relative risk [95% CI] per standard deviation increase in postprandial glycemia was calculated at 1.24 [1.13 to 1.37].

There are multiple mechanisms to explain the link between glycemia and cardiovascular disease including nonenzymatic glycosylation of proteins, generation of free radicals, and upregulation of the polyol and glucosamine pathways [27]. Pharmaceutical interventions to attenuate postprandial glycemia have delayed progression to diabetes and reduced cardiovascular disease events [28, 29]. Recent evidence implies that the peak glucose excursion postmeal is the critical factor for diabetes and cardiovascular disease risk likely because it correlates with the degree of oxidative stress generated [30, 31]. Thus, the reduction in the 60-minute glucose excursion postmeal evoked by liquid vinegar ingestion has important health implications. Indeed, the use of vinegar for blood glucose control is on the rise in the U.S. and globally [32, 33]; yet, consumers should be aware that commercial vinegar tablets when ingested whole may not deliver an antiglycemic effect in the postprandial period similar to liquid vinegar.

## 5. Conclusion

Vinegar has received much attention in recent years as an adjunct therapy for T2D, and empirical evidence is mounting demonstrating its efficacy. These data suggest that although acetic acid content is similar, commercial vinegar tablets taken whole at mealtime are not as effective as liquid vinegar for reducing the postmeal glucose excursion in young, healthy adults.

## Data Availability

The data used to support the findings of this study are available upon request to the corresponding author.
